# Prior hepatitis B virus infection as a co-factor of chronic hepatitis C patient survival after resection of hepatocellular carcinoma

**DOI:** 10.1186/s12876-019-1069-y

**Published:** 2019-08-19

**Authors:** Yutaka Midorikawa, Tadatoshi Takayama, Hisashi Nakayama, Tokio Higaki, Masamichi Moriguchi, Kyoji Moriya, Tatsuo Kanda, Shunichi Matsuoka, Mitsuhiko Moriyama

**Affiliations:** 1 Department of Digestive Surgery, Nihon University School of Medicine, 30-1, Oyaguchikami-cho, Itabashi-ku, Tokyo, 173-8610 Japan; 2 Department of Infectious Diseases, University of Tokyo Faculty of Medicine, Tokyo, Japan; 3 Department of Gastroenterology and Hepatology, Nihon University School of Medicine, Tokyo, Japan

**Keywords:** Prior hepatitis B virus infection, Chronic hepatitis C virus infection, Liver resection, Hepatocellular carcinoma

## Abstract

**Background:**

Prior hepatitis B virus infection (PBI) may increase the risk of developing hepatocellular carcinoma (HCC), but the impact of PBI on clinical outcomes following treatment for HCC remains unknown. The aim of this study was to clarify whether PBI affects clinical outcomes after liver resection for hepatitis C virus (HCV)-related HCC by retrospective cohort study.

**Methods:**

PBI patients were defined as those negative for hepatitis B surface antigen and positive for anti-hepatitis B core antibody. Surgical outcomes of HCV-related HCC patients with PBI were compared to those without PBI. Survival of patients with non-B non-C HCC with and without PBI were also compared.

**Results:**

In the HCV group, the median overall survival of 165 patients with PBI was 4.7 years (95% confidence interval [CI], 3.9–5.9), and was significantly shorter compared with 263 patients without PBI (6.6 years [5.3–9.8]; *p* = 0.015). Conversely, there was no significant difference in recurrence-free survival between the two groups (1.8 years [95% CI, 1.4–2.0] vs 2.0 years [1.7–2.3]; *p* = 0.205). On Cox proportional hazards regression model, independent factors for overall survival were PBI (hazard ratio 1.38 [95% CI, 1.02–1.87]; *p* = 0.033), multiple tumors (*p* = 0.007), tumor size (*p* = 0.002), and liver cirrhosis (*p* <  0.001). On the other hand, in the non-B non-C HCC group, both the median overall survival (6.5 years [95% CI, 4.8–7.1]) and recurrence-free survival (2.4 years, [95% CI, 1.5–3.3]) in 104 patients with PBI were not significantly different from those (7.5 years [5.5 − NA; *p* = 0.932]; and 2.2 years [1.7–2.7; *p* = 0.983]) in 213 patients without PBI.

**Conclusions:**

PBI and HCV in conjunction with each other affect the survival of patients that have undergone resection for HCC.

## Background

Prior hepatitis B virus (HBV) infection (PBI) is evidenced by isolated IgG anti hepatitis B core antibody (HBcAb) positivity, while occult HBV infection (OBI) is defined as the presence of HBV DNA in the liver of patients without hepatitis B surface antigen (HBsAg) [1]. Despite the disappearance of serum HBsAg after spontaneous regression or hepatitis B treatment with nucleoside analogues, for example, some patients are at risk of developing hepatocellular carcinoma (HCC) [2, 3].

PBI or OBI may play a pivotal role in the progression of liver fibrosis in patients with chronic hepatitis C virus (HCV) infection [4–7], and may also be a risk factor for HCC by indirectly causing persistent hepatic inflammation and fibrosis, as well as through its direct proto-oncogenic effect [4, 8, 9]. Consequently, OBI as a co-factor could impact the life-expectancy of chronic HCV patients with OBI compared with those without OBI [9]. However, there are several conflicting reports that indicate there is no association between PBI or OBI and the progression to cirrhosis in chronic HCV patients [10, 11]. Likewise, neither prior exposure to HBV nor OBI are significant factors in HCC development in chronic HCV patients [12–14]. However, there have been a few reports from a small cohort on an association between previous infection of HBV and clinical outcome following treatment for HCC [15, 16]. Thus, the impact of PBI or OBI on clinical outcomes of chronic HCV patients remains controversial and unresolved [17].

Reactivation of HBV, both in prior and overt infection, is characterized by a marked enhancement of viral replication under immunosuppressive conditions [18–20]. Therefore, PBI itself does not seem to exacerbate chronic liver disease preceding hepatocarcinogenesis, but may negatively influence the outcomes of chronic liver disease through the co-existence of other related diseases such as HCV or human immunodeficiency virus infection [21].

The aim of the current study was to demonstrate whether PBI and HCV could co-affect the clinical outcomes of patients undergoing liver resection for HCC.

## Methods

### Patients

From 2004 to 2016, HBsAg-negative patients who underwent curative liver resection for HCC in Nihon University Itabashi Hospital were included in this study. Each participant provided written, informed consent, and this study was approved by the institutional review board of Nihon University. All patients were closely observed during each outpatient visit post-surgery. PBI patients were defined as those who were negative for HBsAg, but positive for HBcAb [22, 23]. HCV patients were defined as those positive for HCV antibody and HCV-RNA detected. Clinical characteristics and outcomes were compared between patients with and without PBI.

### Inclusion and exclusion criteria

Patients who underwent initial liver resection for HCC, but were positive for HBsAg were excluded from the study. Patients who were negative for HBsAg and HBcAb but positive for HBsAb, and those who were positive for HCV antibody but negative for HCV-RNA were also excluded. In addition, patients whose HBsAb or HBcAb status had not been determined were also excluded.

### Indications for liver resection

Indicators for liver resection were determined by assessing the liver functional reserve according to Clinical Practice Guidelines for Hepatocellular Carcinoma in Japan [24]. Briefly, liver resection was contraindicated in patients who had refractory ascites, hepatic encephalopathy, or both [25]. Patients with up to three lesions were candidates for liver resection.

In order to assess the existence of esophageal varices, gastrointestinal endoscopy was performed preoperatively for all candidates considered eligible for liver resection. Patients with F3 varices (largest size) or F2 varices (enlarged tortuous) or blue varices positive for red color signs were treated prophylactically using esophageal variceal ligation [26].

### Surgical procedures

Liver resection was performed on all patients according to criteria based on liver function, as described previously [27]. Briefly, transection of the liver was performed under ultrasonographic guidance using the clamp-crushing method with the inflow-blood-occlusion technique. Curative resection was defined as the complete removal of recognizable HCC diagnosed preoperatively. Postoperative complications were stratified according to the Clavien-Dindo classification [28], which defines morbidities as complications with a score of ≥ IIIa. Complications specific to liver resection were defined as previously described [29].

### Follow-up after surgery

All patients were followed for postoperative recurrence, as described previously [30]. Briefly, tumor marker levels were measured, and imaging studies, including computed tomography and ultrasonography, were performed every 3 months on all patients. Recurrence was diagnosed by dynamic computed tomography and/or gadolinium-ethoxybenzyl-diethylenetriamine pentaacetic acid-enhanced magnetic resonance imaging. The date of recurrence was defined as the date of examination when the recurrence of HCC was detected. In patients with recurrent HCC, the recurrence-free period was defined as the time from the date of surgery to the date of recurrence. Recurrent HCC was managed aggressively by repeated liver resection, transcatheter arterial chemoembolization, radiofrequency ablation, and chemotherapy according to the HCC status and liver function at the time of recurrence. Liver function was estimated 6 months after operation based on Child-Pugh classification.

### Statistical analysis

Data collected from each group were statistically analyzed with Fisher’s exact test and the Wilcoxon rank-sum test. Survival curves were generated using the Kaplan–Meier method and compared by the log-rank test. Prognostic factors for overall survival were identified with the Cox proportional hazards regression model. A *p* value of less than 0.10 was set as the cut-off value for elimination. The following 25 variables considered as potential confounders were examined; age (≥ 75 years vs < 75 years), sex, PBI, alcohol abuse, diabetes mellitus, esophageal varices, AST (≥ 80 U/L vs < 80 U/L), ALT (≥ 80 U/L vs < 80 U/L), platelet count (≥ 10 × 10^4^ μL vs < 10 × 10^4^ μL), creatinine (≥ 1.2 mg/dL vs < 1.2 mg/dL, Child-Pugh classification (A vs B), indocyanine green clearance rate at 15 min (≥ 15% vs < 15%), serum alpha-fetoprotein level (≥ 100 ng/mL vs < 100 ng/mL), serum des-gamma-carboxy prothrombin level (≥ 100 ng/mL vs < 100 ng/mL), operation time (≥ 300 min vs < 300 min), clamp time (≥ 75 min vs < 75 min), bleeding (≥ 300 mL vs < 300 mL), transfusion, resection (anatomic vs non-anatomic), multiple tumors, tumor size (≥ 3.0 cm vs < 3.0 cm), differentiation grade (well-differentiated vs moderately differentiated vs poorly differentiated), tumor thrombus of the portal vein and hepatic vein, tumor exposure at surgery, and liver cirrhosis. In all analyses, a *p* value < 0.05 was considered to be statistically significant.

## Results

### Patients

**Fig. 1:**
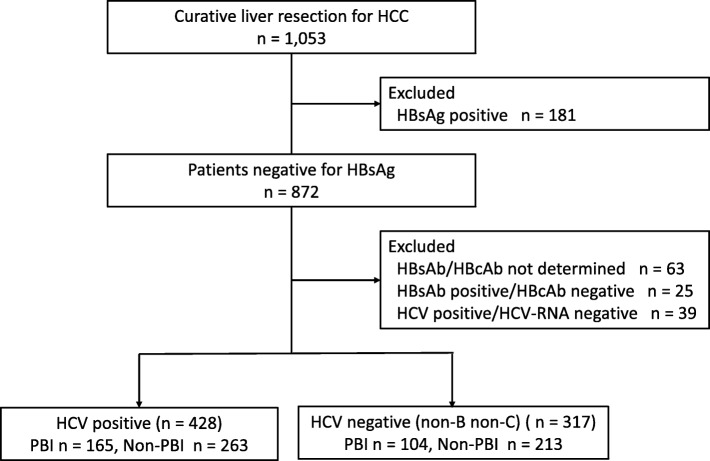
Flow diagram detailing patient recruitment and follow-up study group assignment. The number within each group is indicated (n). HCC, hepatocellular carcinoma; HBsAg, hepatitis B surface antigen; HBsAb, anti-hepatitis B surface antibody; HBcAb, anti-hepatitis B core antibody; HCV, hepatitis C virus; PBI, prior hepatitis B virus infection

Of the 1053 total patients that underwent curative liver resection for HCC during the time period of the study, 872 (82.8%) were negative for HBsAg (Fig. 1). After excluding patients based on the criteria described above, 428 (40.6%) patients, including 165 (18.6%) patients with PBI, were positive for HCV antibody and HCV-RNA; and 317 (30.1%) patients, including 104 (9.8%) patients with PBI were negative for HCV antibody. The demographics and background information for the HCV-positive patients are listed in Table 1. The frequency of alcohol abuse also trended higher in the PBI patients compared to the non-PBI patients (23.6% vs. 16.7%, respectively), but statistical significance was not detected (*p* =  0.080). As seen in Table 2, there was no significant or trending variable between the PBI and non-PBI patients in the group of HCC patients negative for HCV antibody (non-B non-C).

**Table 1:** Patient background (HCV-positive)

	PBI (*n* = 165)	Non-PBI (*n* = 263)	*P*
Age, years	71 (53–85)	70 (32–86)	0.139
Sex, male (%)	121 (73.3)	183 (69.5)	0.444
Alcoholic (%)	39 (23.6)	44 (16.7)	0.080
Diabetes mellitus (%)	40 (24.2)	74 (28.1)	0.560
Varices (%)	48 (29.0)	75 (28.5)	0.912
Platelet count	13.3 (3.2–35.1)	11.7 (3.2–66.0)	0.133
Creatinine	0.75 (0.35–8.34)	9.24 (0.34–9.24)	0.359
Child-Pugh, A (%)	124 (75.1)	204 (77.5)	0.560
ICGR15, %	15.2 (3.6–48.4)	14.8 (2.0–65.5)	0.974
Alpha-fetoprotein, ng/mL	25 (1–23,881)	18 (1–93,075)	0.259
DCP	45 (2–64,386)	44 (1–75,000)	0.591
Operation data			
Operation time, min	331 (139–1004)	300 (120–810)	0.005
Bleeding, mL	316 (5–3887)	240 (10–4530)	0.029
Pringle time, min	72 (0–860)	66 (0–516)	0.162
Transfusion (%)	9 (5.4)	16 (6.0)	0.835
Anatomic resection (%)	57 (34.5)	91 (34.6)	1.000
Complications			
Overall (%)	75 (45.4)	111 (42.2)	0.548
Morbidity (%)	59 (35.7)	93 (35.3)	1.000
Re-operation (%)	5 (3.0)	8 (3.0)	1.000
Mortality (%)	0 (0)	0 (0)	1.000
Pathology			
Multiple (%)	51 (30.9)	67 (25.4)	0.223
Size, cm	2.8 (0.7–12.5)	2.5 (0.7–18.0)	0.171
Differentiation grade			
well/moderately/poorly	36/114/15	59/175/29	0.808
Vascular invasion (%)	39 (23.6)	74 (25.8)	0.648
Tumor exposure (%)	11 (6.6)	23 (8.7)	0.469
Cirrhosis (%)	75 (45.4)	133 (50.5)	0.321

Data are presented as median with range, if not specified

*HCV* Hepatitis C virus, *PBI* Prior hepatitis B virus infection, *ICGR15* Indocyanine green clearance rate at 15 min, *DCP* Des-gamma carboxyprothrombin

**Table 2:** Patient background (non-B non-C)

	PBI (*n* = 104)	Non-PBI (*n* = 213)	*P*
Age, years	70 (44–85)	69 (36–84)	0.250
Sex, male (%)	86 (82.6)	177 (83.0)	1.000
Alcoholic (%)	45 (43.2)	82 (38.4)	0.464
Diabetes mellitus (%)	50 (48.0)	96 (45.0)	0.632
Varices (%)	18 (17.3)	33 (15.4)	0.745
Platelet count	18.0 (4.3–66.5)	17.7 (3.7–44.3)	0.851
Creatinine	0.78 (0.35–2.44)	0.78 (0.36–2.37)	0.448
Child-Pugh, A (%)	102 (90.3)	190 (89.2)	0.846
ICGR15, %	10.6 (2.3–48.0)	11.8 (1.3–74.6)	0.254
Alpha-fetoprotein, ng/mL	7 (1–145,900)	7 (1–39,596)	0.578
DCP	126 (6–75,000)	142 (8–75,000)	0.553
Operation data			
Operation time, min	325 (130–609)	334 (97–855)	0.888
Bleeding, mL	261 (10–2398)	384 (5–2065)	0.133
Pringle time, min	71 (12–222)	67 (0–485)	0.309
Transfusion (%)	7 (6.7)	24 (11.2)	0.232
Anatomic resection (%)	44 (42.3)	87 (40.8)	0.809
Complications			
Overall (%)	30 (29.2)	70 (32.8)	0.520
Morbidity (%)	26 (25.0)	53 (24.8)	0.981
Re-operation (%)	4 (3.8)	4 (1.8)	0.446
Mortality (%)	0 (0)	0 (0)	1.000
Pathology			
Multiple (%)	26 (25.0)	54 (25.3)	1.000
Size, cm	4.0 (1.0–15.5)	0.9 (4.3–20.5)	0.438
Differentiation grade			
well/moderately/poorly	14/77/13	35/156/22	0.709
Vascular invasion (%)	35 (33.6)	81 (38.0)	0.459
Tumor exposure (%)	12 (11.5)	13 (6.5)	0.119
Cirrhosis (%)	22 (21.1)	45 (21.1)	1.000

Data are presented as median with range, if not specified

*PBI* Prior hepatitis B virus infection, *ICGR15* Indocyanine green clearance rate at 15 min, *DCP* Des-gamma carboxyprothrombin

### Operative data

Among the patients positive for HCV antibody and HCV-RNA, the amount of bleeding during surgery for PBI patients (median 316 mL; range, 5–3887) was significantly greater compared to that of non-PBI patients (240 mL; 10–4530; *p* = 0.029) (Table 1). In comparison, there was no significant variable between the PBI and non-PBI patients negative for HCV (Table 2). Complication rates and histological diagnosis were also not significantly different between the two PBI groups in both HCV-positive and HCV-negative patients.

### Survival of HCV-positive patients

After a median follow-up of 3.6 years (range, 0.2–14.2), a total of 290 patients (67.7%) experienced HCC recurrence; 277 patients (95.5%) in the remnant liver, eight patients (2.7%) in distant sites such as the lung, bone, and lymph nodes, and five patients (1.7%) with both intra- and extra-hepatic recurrences. The treatments for recurrent HCC were not significantly different between the two groups (Table 3). During the follow-up period, 105 (63.6%) patients with PBI and 190 (72.2%) patients without PBI were diagnosed as Child-Pugh classification A (*p* = 0.068).

**Table 3:** Treatment after recurrence (HCV positive)

Treatments	PBI (*n* = 117)	Control (*n* = 173)	*P*
Resection^a^	48 (41.0)	65 (37.5)	0.623
Transcatheter arterial chemoembolization	58 (49.5)	96 (55.4)	0.339
Radiofrequency ablation	3 (2.5)	4 (2.3)	1.000
Chemotherapy	4 (3.4)	4 (2.3)	0.718
Radiation	1 (0.8)	2 (1.1)	1.000
None	3 (2.5)	2 (1.1)	0.396

*HCV* Hepatitis C virus, *PBI* Prior hepatitis B virus infection

^a^including resection for extra-hepatic tumors

**Fig. 2:**
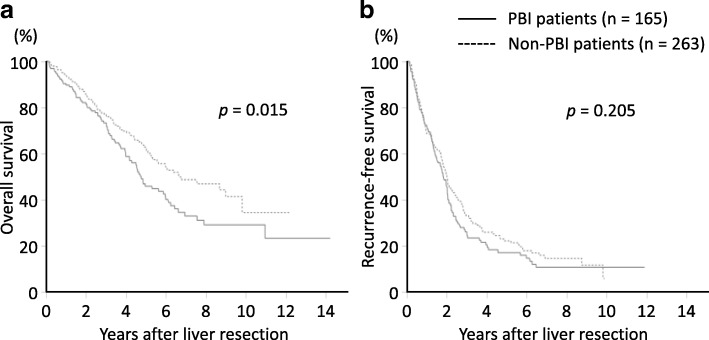
Survival outcomes following liver resection in HCV patients. **a** The overall survival of patients with prior HBV infection (PBI) was significantly shorter compared to those without PBI (*p* = 0.015). **b** The recurrence-free survival was not significantly different between the two groups (*p* = 0.205). Study group sizes are indicated (n)

In the patients with HCV-positive, the median overall survival of PBI patients was 4.7 years (95% confidence interval [CI], 3.9–5.9), which was significantly shorter compared with that of non-PBI patients (6.6 years, 5.3–9.8; *p* = 0.015) (Fig. 2). On the other hand, there was no significant difference in the median recurrence-free survival between the two groups (1.8 years, [95% CI, 1.4–2.0] vs 2.0 years, 1.7–2.3; *p* = 0.205). The overall survival and recurrence-free survival rates at 5 years were 45.9 and 17.0% in PBI patients, respectively, and 62.0 and 22.1% in non-PBI patients, respectively. On multivariate analysis, independent factors for overall survival were PBI (hazard ratio 1.38 [95% CI, 1.02–1.87]; *p* = 0.033), multiple tumors (*p* = 0.007), tumor size (*p* = 0.002), and liver cirrhosis (*p* <  0.001) (Table 4).

**Table 4:** Prognostic factors for survival in patients with HCV

Variables	Univariate		Multivariate	
	Odds ratio	*p*	Odds ratio	*p*
Age	1.16 (0.78–1.68)	0.446		
Gender, male	0.79 (0.54–1.14)	0.215		
Prior HBV infection	1.45 (1.06–1.99)	0.019	1.38 (1.02–1.87)	0.033
Alcoholic	0.82 (0.54–1.22)	0.348		
Diabetes mellitus	1.05 (0.72–1.51)	0.755		
Varices	1.05 (0.74–1.49)	0.747		
AST	0.93 (0.61–1.37)	0.747		
ALT	0.69 (0.45–1.03)	0.171		
Platelet count	0.84 (0.62–1.16)	0.303		
Creatinine	1.46 (0.72–2.62)	0.242		
Child-Pugh, B	1.17 (0.79–1.72)	0.413		
ICGR15	1.05 (0.74–1.48)	0.762		
Alpha-fetoprotein	0.91 (0.62–1.29)	0.617		
DCP	1.22 (0.87–1.72)	0.241		
Operation time	1.04 (0.70–1.54)	0.822		
Pringle time	0.88 (0.60–1.29)	0.513		
Bleeding	0.91 (0.64–1.28)	0.606		
Transfusion	1.02 (0.50–1.87)	0.947		
Anatomic resection	0.90 (0.63–1.27)	0.570		
Multiple	1.76 (1.23–2.50)	0.002	1.53 (1.12–2.08)	0.007
Size	1.55 (1.10–2.20)	0.012	1.62 (1.18–2.22)	0.002
Differentiation grade	1.45 (0.99–2.17)	0.517		
Vascular invasion	1.38 (0.96–1.96)	0.077	1.34 (0.94–1.86)	0.094
Tumor exposure	1.33 (0.74–2.24)	0.313		
Cirrhosis	1.50 (1.07–2.12)	0.018	1.68 (1.24–2.29)	< 0.001

*HCV* Hepatitis C virus infection, *HBV* Hepatitis B virus infection, *AST* Aspartate aminotransferase, *ALT* Alanine aminotransferase, *ICGR15* Indocyanine green clearance rate at 15 min, *DCP* Des-gamma carboxyprothrombin

### Survival of non-B non-C patients

After a median follow-up of 3.0 years (range, 0.2–12.8 years) for the non-B non-C patients in the study, a total of 192 patients (60.5%) experienced HCC recurrence; 160 patients (83.3%) in the remnant liver, 22 patients (11.4%) in distant sites, and 10 patients (5.2%) with both intra- and extra-hepatic recurrences.

**Fig. 3:**
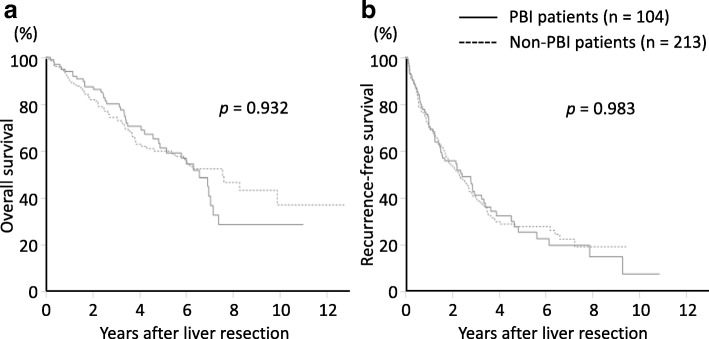
Survival outcomes following liver resection in non-B non-C patients. **a** The overall survival of patients with prior HBV infection (PBI) was not significantly different compared to those without PBI (*p* = 0.932) **b** The recurrence-free survival was not significantly different between the two groups (*p* = 0.983). Study group sizes are indicated (n)

For the 104 patients with PBI and the 213 patients without PBI in this group, the median overall survival was 6.5 years (95% CI, 4.8–7.1) and 7.5 years (5.5–NA; *p* = 0.932), respectively. The recurrence-free survivals were 2.4 years (95% CI, 1.5–3.3) and 2.2 years (1.7–2.7; *p* = 0.983), respectively (Fig. 3). The 5-year overall survival rates were 61.2 and 59.9%, and 5-year recurrence-free survival rates were 25.3 and 27.7% in the two groups, respectively.

## Discussion

We found that PBI was an unfavorable prognostic factor, negatively impacting survival rates following liver resection for HCV-related HCC, while it did not affect the surgical outcomes in non-B non-C HCC patients. Therefore, our findings suggest that PBI and HCV in conjunction with each other affect the survival of patients that have undergone resection for HCC.

Consistent with the previous report that HCV and OBI co-infected patients are at an increased risk of lower survival [9], we showed that overall survival following resection for HCC was significantly shorter in HCV patients with PBI compared with those without PBI. On the other hand, no association between PBI and survival was observed in non-B non-C HCC patients, which strongly suggests that PBI by itself is not a significant factor. However, when other significant immunosuppressive causes of liver damage co-exist such as HCV [31], human immunodeficiency virus infection [32], and immunosuppression therapy [18, 20], PBI or OBI may contribute to a worsening of the course of chronic liver disease. In this study, liver function trended to be worse in PBI patients with HCV-positive, which could account for the poorer overall survival of such patients despite of no significant difference of recurrence-free survival between the HCV-positive patients with and without PBI. Furthermore, surgical stress, as well as HCV infection, might also accelerate the deteriorative outcomes of PBI patients undergoing resection of HCC.

Compared with non-B non-C patients with PBI, overall survival rate at 5 years for HCV patients with PBI in this study was lower due to the high frequency of cirrhosis (advanced fibrosis), Child-Pugh classification B (poor liver function), and varices (portal hypertension). On the other hand, despite of the poorer liver function, survival rates of HCV patients without PBI were similar to those of non-B non-C patients without PBI, which also supports the idea that there may be some negative synergistic effects on the survival of patients after resection of HCC between PBI and HCV infections [31].

It is debatable whether PBI or OBI plays a pivotal role in the progression of fibrosis in HCV patients [5, 7, 10, 11]. In our study, the prevalence of patients with cirrhosis in the PBI group was not statistically different from those in the non-PBI group. Similarly, there was no association between PBI and the progression of cirrhosis in non-B non-C HCC patients, suggesting that PBI was not a predictor for cirrhosis, regardless of HCV-infection status. However, it should be noted that the population in this study was limited to candidates for surgery, and the results may not be simply comparable to that of previous studies.

Despite the numerous reports about the impact of PBI or OBI on HCV infection, there is only one report to date that analyzed survival after resection for patients with HCV-related HCC in association with PBI [15]. In contrast to our findings, they reported that the overall survival was not significantly different between the patients with and without PBI. This discrepancy may be attributed to the significantly higher frequency of anatomic resection in the HBcAb-positive group (69.7% vs 50.0%), despite similar liver function of the two groups. This might initiate the negative effects of PBI, and the positive effects of anatomic resection on survival.

On the other hand, the comparative study reported by Wu et al. focused on OBI in patients with non-B non-C HCC [16]. The period of recurrence-free survival was significantly longer, and the overall survival trended to be longer. Conversely, in another report by Itoh and colleagues, there was no significant differences in overall or recurrence-free survivals between non-B non-C HCC patients with and without PBI [15]. This is due to a higher recurrence-free survival rate at 5 years for patients without OBI (about 50%) in the former report [16], although overall and recurrence-free survival of patients with OBI are consistent with our data. In any case, there were a relatively small number of participants in their studies, and large-scale studies should be performed to justify these procedures in the future.

## Conclusions

Our data that PBI negatively influenced the clinical outcomes in HCC patients with HCV infection that underwent HCC resection, but not in those without HCV. This might be attributed to the concept that PBI works as a co-effector with HCV to impact the outcomes for HCC patients.

## Data Availability

Data and materials analyzed in this study are not available due to the pretection of individual privacy, but are available from the corresponding author on reasonable request.

## Abbreviations

HBcAb: Anti-hepatitis B core antibody
HBsAb: Anti-hepatitis B surface antibody
HBsAg: Hepatitis B surface antigen
HBV: Hepatitis B virus
HCC: Hepatocellular carcinoma
HCV: Hepatitis C virus
OBI: Occult hepatitis B virus infection
PBI: Prior hepatitis B virus infection
